# An Order of Magnitude Faster AIP1-Associated Actin Disruption than Nucleation by the Arp2/3 Complex in Lamellipodia

**DOI:** 10.1371/journal.pone.0004921

**Published:** 2009-03-17

**Authors:** Takahiro Tsuji, Takushi Miyoshi, Chiharu Higashida, Shuh Narumiya, Naoki Watanabe

**Affiliations:** Department of Pharmacology, Kyoto University Faculty of Medicine, Kyoto, Japan; University of Birmingham, United Kingdom

## Abstract

The mechanism of lamellipod actin turnover is still under debate. To clarify the intracellular behavior of the recently-identified actin disruption mechanism, we examined kinetics of AIP1 using fluorescent single-molecule speckle microscopy. AIP1 is thought to cap cofilin-generated actin barbed ends. Here we demonstrate a reduction in actin-associated AIP1 in lamellipodia of cells overexpressing LIM-kinase. Moreover, actin-associated AIP1 was rapidly abolished by jasplakinolide, which concurrently blocked the F-actin-cofilin interaction. Jasplakinolide also slowed dissociation of AIP1, which is analogous to the effect of this drug on capping protein. These findings provide *in vivo* evidence of the association of AIP1 with barbed ends generated by cofilin-catalyzed filament disruption. Single-molecule observation found distribution of F-actin-associated AIP1 throughout lamellipodia, and revealed even faster dissociation of AIP1 than capping protein. The estimated overall AIP1-associated actin disruption rate, 1.8 µM/s, was one order of magnitude faster than Arp2/3 complex-catalyzed actin nucleation in lamellipodia. This rate does not suffice the filament severing rate predicted in our previous high frequency filament severing-annealing hypothesis. Our data together with recent biochemical studies imply barbed end-preferred frequent filament disruption. Frequent generation of AIP1-associated barbed ends and subsequent release of AIP1 may be the mechanism that facilitates previously observed ubiquitous actin polymerization throughout lamellipodia.

## Introduction

Motile cells extend dynamic fan-shaped pseudopods called lamellipodia at the leading edge. In lamellipodia, filamentous actin (F-actin) continuously moves inward along the retrograde flow [1], [2]. To balance with constant clearance of F-actin, the most plentiful filament assembly occurs at the lamellipodium tip [3], [4]. F-actin assembly and disassembly also occur throughout lamellipodia. This ubiquitous F-actin turnover has initially been revealed by the fast disintegration of the actin network observed using photoactivatable actin [5]. Our single-molecule observation of fluorescent actin has confirmed it and further elucidated detailed F-actin lifetime distribution in lamellipodia [6]. Notably, one third of newly-assembled F-actin had a short lifetime of less than 10 sec. These observations highlight the dynamic remodeling of the actin network in the body of lamellipodia. Subsequent computational analysis of fluorescent speckle microscopy also detected largely overlapping actin assembly and disassembly activities within lamellipodia (see Fig. 1F in [7]). On the other hand, a recent work employing actin FRAP (fluorescence recovery after photobleaching) [8] shows slow recovery of photobleached labels, and the authors of this study argue against our conclusion of fast actin turnover throughout lamellipodia.

**Figure 1:**
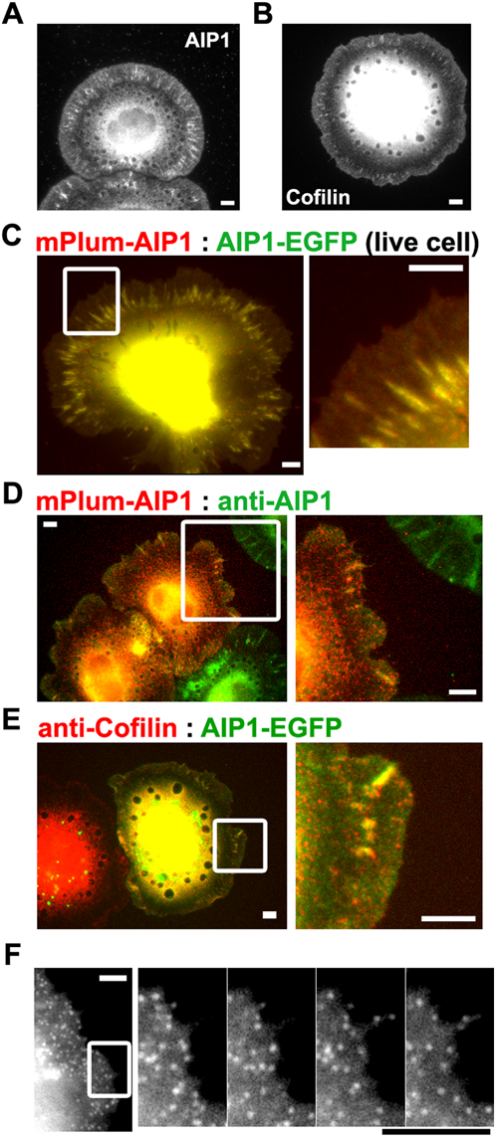
Colocalization of AIP1 probes with cofilin and endogenous AIP1 in XTC cells. (A and B) Distribution of AIP1 and cofilin in XTC cells. Cells were fixed 45 min after seeded on poly-L-lysine (PLL)-coated glass coverslips and processed for immunostaining. (C) mPlum-AIP1 (red) and AIP1-EGFP (green) displayed identical distribution in live XTC cells. A part of the left image (square) is enlarged on the right. Rapidly moving red signals in the lamellae region (see also Supplementary Movie S1) presumably arise from mPlum-AIP1 degraded and retained in cell organelles, which we often observe with constructs tagged with mPlum, mRFP1 or mCherry but not with EGFP. (D) Comparison between endogenous AIP1 (green) and mPlum-AIP1 (red) in fixed cells. (E) Comparison between endogenous cofilin (red) and AIP1-EGFP (green). A part of left images (squares) are enlarged on the right (D and E). (F) Images of AIP1-EGFP speckles in lamellipodia. Images of cells expressing a low level of AIP1-EGFP were acquired. Enlarged timelapse images paneled at 2 sec intervals in the area (square) are shown on the right. Bars, 5 µm.

Actin disassembly to monomer recycling remains one of the most obscurely understood steps in the actin turnover cycle although the mechanism of lamellipod extension has attracted increasing interest of researchers since the discovery of dendritic nucleation by Arp2/3 complex [9], [10], [11]. Filament severing is another measure for the generation of barbed ends in addition to nucleation. A number of attempts have been made to quantitatively model actin-driven lamellipod extension. Despite accumulating evidence by us [12] and others [13], however, filament severing and end-to-end annealing have been neglected in recent theoretical studies [14], [15], [16]. This may be due to the lack of knowledge regarding the spatiotemporal regulation of severing and its controlling mechanisms such as filament length distribution [17] and the activity of cofilin [18], [19]. A recent study [20] demonstrating promotion of filament nucleation by a high concentration of cofilin refutes its ‘slow and stoichiometric (non-catalytic)’ severing activity [21] as the primary function of cofilin. However, in cells, studies showing a marked reduction in the actin disassembly rate by inactivation of cofilin [12], [22], [23] provide evidence for an essential role of cofilin in actin disassembly.

Our recent study [12] has revealed data implying fast F-actin severing in lamellipodia. Capping protein (CP) observed at the single-molecule level dissociates approximately 25-fold faster than actin disassembly. This fast CP dissociation is specific to *in vivo* because our EGFP-tagged CP probe tightly caps the barbed end with a dissociation constant of 1.3 nM *in vitro* [12]. Notably, F-actin stabilizing treatments such as jasplakinolide (Jas) and overexpression of LIMK1, which inactivates cofilin [24], strongly attenuate the dissociation of CP. CP speckles associating with actin stress fibers showed slower dissociation kinetics than those in the other regions. These findings suggest that cofilin-mediated F-actin severing/disruption triggers CP dissociation from F-actin. The marked difference between the CP dissociation and the actin disassembly rates led us to hypothesize that filament severing as well as end-to-end annealing might take place frequently [12]. If we assume a uniform filament severing rate throughout the actin network, our finding implies a frequent filament severing and annealing rate on the order of 1 s^−1^ per ∼30 subunit long filaments.

In the present study, we aimed to estimate the frequency and distribution of cofilin-mediated filament disruption in lamellipodia. As a probe for fluorescence single-molecule observation, we used AIP1 (Actin interacting protein 1; reviewed in [25]). AIP1 shows genetic interaction with cofilin in *yeast* [26], [27] and *C. elegans* [28], in which they collaborate to disassemble F-actin [29], [30]. In *mouse*, two amino acid deletion in AIP1 causes neutrophil dysfunction and macrothrombocytopenia [31]. The combination of cofilin and AIP1 induces strong fragmentation of actin filaments [32], [33]. AIP1 caps the barbed end in a manner dependent on cofilin and inhibits elongation from the barbed end generated by cofilin [34]. In contrast, AIP1 does not prevent reannealing of mechanically fragmented filaments [34]. Hence AIP1 specifically recognizes the barbed end generated from filament severing/disruption catalyzed by cofilin. More recent studies have revealed that the addition of coronin enables cofilin and AIP1 to achieve fast actin disassembly in a manner insensitive to polymerizable monomer [35]. This three component system facilitates actin disassembly from both barbed and pointed ends by abruptly removing filaments of a mean size of 260 subunits [36]. It is currently unknown whether this instantaneous subunit loss along the length of filaments is achieved by cooperative strand separation [36] or by filament-end severing. Nevertheless, this mechanism involving AIP1 and cofilin seems to be the best candidate for the cellular actin disassembly mechanism in terms of its potential to facilitate filament disassembly fast enough to account for the cellular actin dynamics. During actin disassembly induced by the three proteins, new barbed ends are protected from CP [36]. Therefore, whatever the mechanism is, biochemical evidence supports our use of AIP1 for monitoring cofilin-mediated filament disruption/severing and barbed end formation.

Here, using single-molecule observation, we first demonstrate *in vivo* evidence that AIP1 caps the barbed end generated by cofilin-mediated filament disruption/severing. Supported by these notions, our quantitative analysis reveals the substantially faster formation of AIP1-associated barbed ends than Arp2/3 complex-catalyzed actin nucleation in lamellipodia, although the overall frequency of AIP1-associated F-actin disruption/severing is less than the high frequency severing rate we previously postulated [12]. We discuss possible mechanisms for this discrepancy including filament end-preferred actin disruption/severing which has recently been demonstrated *in vitro* [36]. In addition, we briefly discuss technical problems that may account for apparent discrepancy between single-molecule observation [6] and FRAP analysis [8]. Our findings of fast association-dissociation kinetics of barbed end factors ([12], this study) reveal vigorous actin remodeling through filament disruption/severing in the body of lamellipodia.

## Results

### Colocalization of EGFP-tagged AIP1 probes with cofilin and endogenous AIP1

To generated fluorescent probes of AIP1, we tagged either the N- or C-terminus of AIP1 of *Xenopus* origin with an enhanced green fluorescent protein (EGFP) or a monomeric red fluorescent protein, mPlum [37]. We first show the localization of endogenous AIP1 and cofilin in XTC *Xenopus* fibroblast cells spreading on the poly-L-lysine (PLL)-coated glass coverslips (Fig. 1A and B). AIP1 and cofilin displayed similar association to the F-actin meshwork in lamellipodia (see also [37]). Filopodium bundles were stained weakly with antibodies against AIP1 and cofilin while actin stress fibers were not labeled. A previous study found that cofilin is excluded from a distal 0.4 µm wide lamellipodium tip in keratocytes [11]. In contrast, cofilin and AIP1 distributed throughout lamellipodia of XTC cells. This is in agreement with our previous observations of the slight increase in F-actin lifetime at the lamellipodium tip of XTC cells [6]. We also noted that AIP1 is enriched in F-actin clusters found at the base of lamellipodia, where actin nucleation by mDia1, a Formin protein, is locally promoted through increased release of actin monomers [38]. Both N- and C-terminally tagged AIP1 probes displayed identical subcellular localization (Fig. 1C, Movie S1), associating with the actin network in lamellipodia. These fluorescent protein-tagged AIP1 probes display similar localization with endogenous AIP1 (Fig. 1D) and cofilin (Fig. 1E). These results confirm that two AIP1 constructs can be used as reliable probes for monitoring intracellular behavior of AIP1. We used both N- and C-terminally tagged AIP1 probes in the following single-molecule speckle analysis.

In cells expressing a low level of EGFP-tagged AIP1, images appeared as discrete spots of fluorescence speckles (Fig. 1F, Movie S2). AIP1 speckles were enriched in lamellipodia and moved inward along the retrograde actin flow. Fluorescence intensity of individual isolated speckles was comparable to that of single-molecule actin and CP speckles [6], [12], suggesting that the isolated AIP1 speckles consist of a single EGFP-tagged molecule. As shown previously [6], formation of speckle signals depends on immobilization of fluorescent probes onto cellular structures. Together with its co-distribution with F-actin in lamellipodia (Fig. 1C–E), we interpret that the formation of speckle images represent AIP1 molecules interacting with F-actin.

### Association of AIP1 with cellular F-actin depends on cofilin activity

**Figure 2:**
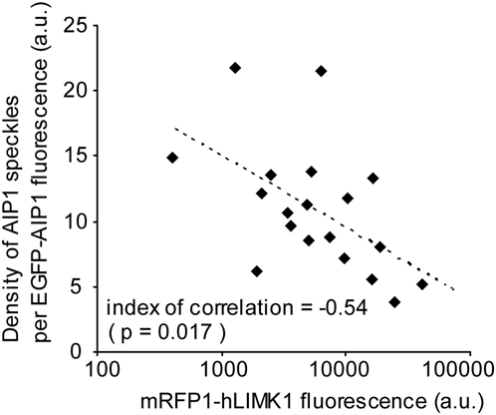
Overexpression of mRFP1-hLIMK1 abrogates the formation of AIP1 speckles. XTC cells were trasfected with various ratios of the expression vectors for AIP1-EGFP and mRFP1-hLIMK1. Fluorescence images of live cells expressing AIP-EGFP at very low levels were acquired 0.5–1 hr after cell spreading onto PLL-coated glass coverslips. The degree of AIP1-actin association was calculated by dividing the number of single-molecule AIP1 speckles in lamellipodia by the total EGFP fluorescence in the measured area. The mRFP1-hLIMK1 fluorescence indicates the total mRFP1 fluorescence in the entire cell area. The data show negative correlation between the normalized speckle density of AIP1 and the expression level of mRFP1-hLIMK1. Pearson product-moment correlation coefficient: *r* = −0.54, *N* = 19 cells, *p* = 0.017.

AIP1 bind the barbed end only in the presence of cofilin and does not prevent reannealing of mechanically fragmented F-actin [34]. Therefore, AIP1 is thought to recognize the barbed end of cofilin-bound filaments. We tested whether the formation of AIP1 speckles depends on the activity of cofilin in live cells. We previously employed overexpression of mRFP1-hLIMK1 for depleting cofilin activity. Overexpressed mRFP1-hLIMK1 attenuates actin disassembly as well as the dissociation of CP from F-actin in lamellipodia [12]. In the current study, we examined the effect of mRFP1-hLIMK1 overexpression on the degree of F-actin association of AIP1. To measure the degree of F-actin association, we first selected cells expressing a very low level of AIP1-EGFP, which was suited for single-molecule counting, along with various amounts of mRFP1-hLIMK1 for the measurement. We counted the number of single-molecule AIP1 speckles in lamellipodia, and then divided this number by the integrated fluorescence intensity of AIP1-EGFP in the measured area. This yields a relative degree of the ratio of F-actin associated molecules to the whole population of AIP1-EGFP existing in the area. This normalized density of single-molecule AIP1 speckles was negatively correlated with the expression level of mRFP1-hLIMK1 (Fig. 2). These results provide *in vivo* evidence that the association of AIP1 with F-actin is dependent on the activity of cofilin.

### Rapid, concurrent inhibition of F-actin binding of AIP1 and cofilin by jasplakinolide

The above data demonstrate the dependency of the interaction between F-actin and AIP1 on the activity of cofilin. Given the strong impact of LIMK1 overexpression on F-actin turnover [12], [22], [23], however, the reduction of actin-associated AIP1 could be caused secondarily by long-term deterioration of actin dynamics and/or abnormal organization of the actin network in LIMK1-overexpressing cells. We therefore sought another treatment that can perturb the activity of cofilin acutely.

We have previously found that an actin depolymerization inhibitor, jasplakinolide (Jas) strongly attenuates dissociation of CP from actin. Jas, binds F-actin competitively with another F-actin stabilizing drug, phalloidin [39], and probably exerts anti-severing effects against cofilin in a manner similar to phalloidin. We then interpreted that attenuation of CP dissociation was caused by inhibition of cofilin-catalyzed filament severing by Jas [12].

**Figure 3:**
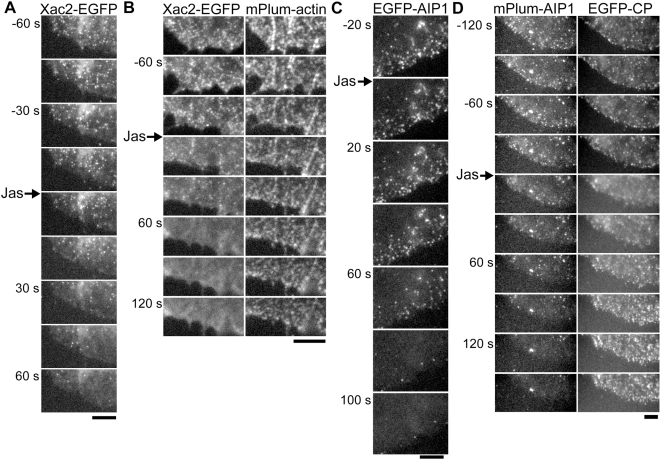
Jasplikinolide rapidly blocks appearance of cofilin and AIP1 speckles. (A) Disappearance of XAC2-EGFP speckles induced by perfusion of 4 µM jasplakinolide (Jas). (B) XAC2-EGFP speckles disappear quickly after 4 µM Jas treatment (left panels), whereas actin speckles and F-actin structures are well preserved. (C) Rapid disappearance of EGFP-AIP1 speckles after perfusion of 1 µM Jas. (D) AIP1 speckles disappear quickly after the treatment with 4 µM Jas (left panels), whereas capping protein (CP) speckles remain persistently (right panels). As shown in the previous study (Miyoshi et al., 2006), a marked prolongation of retention time of CP speckles is observed. The loss in EGFP and mPlum signals due to photobleaching is less than 15% and 50%, respectively. Bars, 5 µm.

Here we demonstrate direct evidence that Jas interferes with the cofilin-actin interaction in cells. We generated a GFP-tagged cofilin probe of XAC2 (XAC2-EGFP), one of two closely-related cofilin isoforms in *Xenopus laevis* [40]. We tagged the C-terminus of XAC2 with EGFP. Interestingly, XAC2-EGFP appeared as speckles in lamellipodia when expressed at a low level (Fig. 3A and B). Cofilin speckles showed similar localization with AIP1, distributed throughout the lamellipodial F-actin network. Upon perfusion of Jas, we observed rapid disappearance of cofilin speckles within a minute (Fig. 3A). Cofilin speckles disappeared before collapse of the F-actin network as judged by persistent speckles of coexpressed mPlum-actin (Fig. 3B). Although the precise function of F-actin-attached cofilin visualized as speckle images is unknown, our data clearly show that Jas interferes with the interaction of cofilin with F-actin in cells.

We next examined the effect of Jas on the behavior of AIP1 speckles. Notably, AIP1 speckles also disappeared quickly upon perfusion of Jas (Fig. 3C). The time course of the loss in the speckle formation was similar between cofilin and AIP1. In contrast, CP speckles persisted for the extended duration after Jas treatment (Fig. 3D) as described in our previous study [12]. Thus the interaction between AIP and F-actin was abolished by Jas despite the presence of barbed ends which CP can bind. Together with the decrease in the density of AIP1 speckles induced by LIMK1 overexpression (Fig. 2), these results provide *in vivo* evidence that the interaction of AIP1 with F-actin is dependent on the interaction of cofilin with F-actin.

### Jasplakinolide slows dissociation of AIP1: analogous effect on CP

Previously we found marked prolongation of CP speckle retention time upon stabilization of F-actin induced by Jas. We interpret these observations as showing the dependency of CP dissociation on F-actin severing [12]. If this interpretation is correct, dissociation of other barbed end cappers including AIP1 ought to be attenuated by F-actin stabilization.

**Figure 4:**
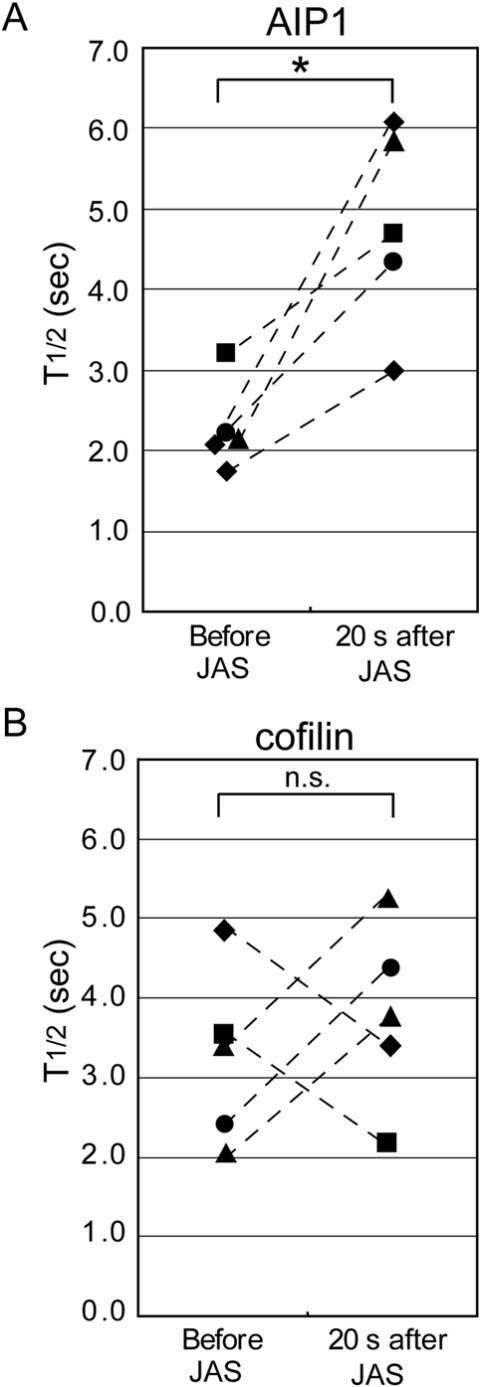
Dissociation of AIP1, but not of cofilin, slows by jaskplakinolide treatment; analogous to Jas-induced stabilization of capping protein. Images of EGFF-AIP1 (A) or XAC2-EGFP (B) speckles in live XTC cells were acquired before and 20–40 s after treatment with 2 µM jasplakinolide. The decay rate of persistent single-molecule AIP1 or cofilin speckles before and 21–34 s after drug perfusion is compared in the same cells (connected by dashed lines) and expressed as half-life (n = 5 cells for each construct). *p = 0.012 (A), n.s.: not significant (B), paired *t*-test, two-tailed.

To test this, we acquired single-molecule AIP1 images before and shortly after perfusion of Jas and compared the decay rate of persistent AIP1 speckles. As described above (Fig. 3), Jas eliminated the formation of AIP1 speckles completely ∼1 minute after drug perfusion. However, before their complete loss, AIP1 speckles temporally showed significantly slower dissociation kinetics 20–40 sec after drug administration than prior to the treatment (Fig. 4A). These results indicate that in addition to its strong inhibitory effect on *de novo* association of AIP1 to F-actin, Jas attenuates dissociation of AIP1 bound to the barbed end. *In vitro*, AIP1 cosediments with F-actin with low affinity (estimated dissociation constant of 15 µM) [32]. This rapidly dissociating side-binding of AIP1 would not be detected using our current experiment setting. By analogy to the strong inhibitory effect of Jas on dissociation of CP from actin [12], we conclude that AIP1 speckles represent their association with the actin barbed end.

We next examined whether Jas might also attenuate the dissociation of cofilin from the actin network. We compared the decay rates of persistent cofilin (XAC2-EGFP) speckles before and shortly after Jas treatment (Fig. 4B). No apparent change in the decay rate of cofilin speckles was induced upon Jas perfusion.

It must be mentioned that the Jas-induced increase in the half-life of persistent AIP1 speckles (1.5–2.9 fold) is much smaller than that of CP (>15 fold). This is probably because of the weaker affinity of AIP1 to the barbed end than that of CP. CP binds to barbed ends with 0.1–1 nM dissociation constant and the off-rate of CP from barbed ends is ∼0.0004 s^−1^ *in vitro* [41], [42]. Although the precise affinity of AIP1 with the barbed end is unknown, AIP1 appears to bind the barbed end with a dissociation constant of submicromolar ranges [34]. New barbed ends formed during the burst mode actin disassembly by cofilin, coronin and AIP1 are protected against CP by AIP1, which dissociates rapidly from the barbed end (τ<10 s) [36]. AIP1 may therefore dissociate faster than CP in the absence of the severing-triggered release mechanism.

### Lifetime distribution of single-molecule AIP1 probes in lamellipodia

**Figure 5:**
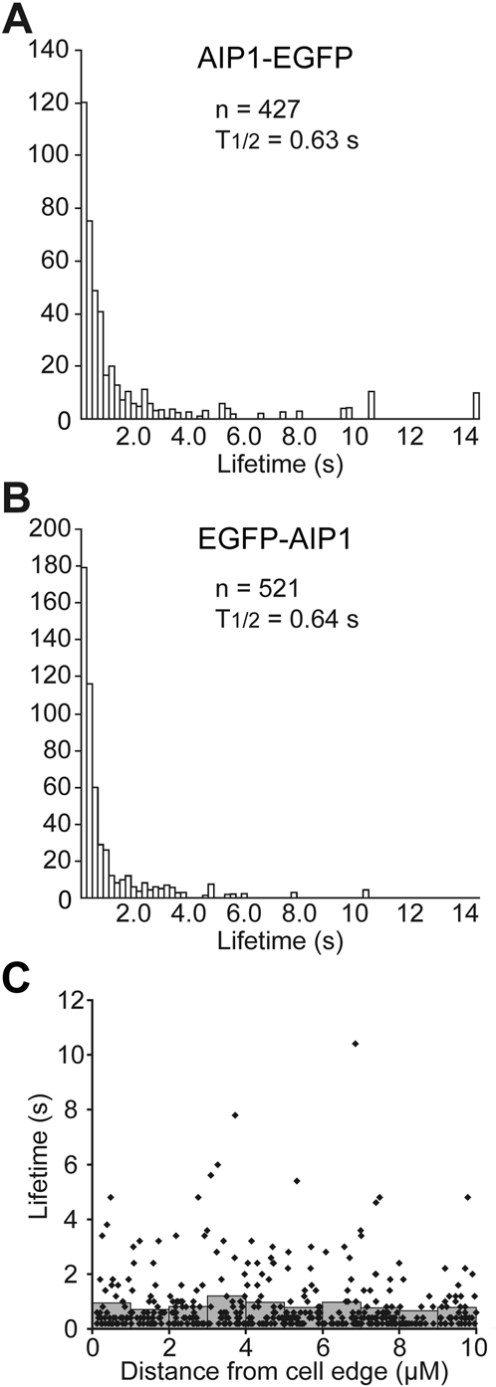
Lifetime distribution of single-molecule AIP1 speckles in lamellipodia. (A and B) Speckle images in lamellipodia of XTC cells expressing a low amount of EGFP-AIP1 or AIP1-EGFP were acquired at intervals of 200 ms. The graphs show speckle lifetime distribution of AIP1-EGFP, n = 427, 1 cell (A) and EGFP-AIP1, n = 521, 1 cell (B), respectively. A representative result from 3 measurements is shown for each construct. Speckles with single EGFP intensity which appeared over the course of a 3 s time window were followed. Columns show the number of speckles with indicated lifetime after normalization for photobleaching. The decay rate is expressed as half-life (T_1/2_) which was calculated by fitting the lifetime data between 0.4 and 3 s with a single exponential. The dissociation rate of AIP1 is faster than that of capping protein (T_1/2_ = 1.20–1.23 s) observed previously [12]. (C) Lifetime versus position plot of AIP1 speckle lifetime data. Dots indicate lifetime and emerging position of individual EGFP-AIP1 speckles. Gray columns, the average speckle lifetime in each indicated area after normalization for photobleaching.

To estimate the association-dissociation frequency of AIP1 on the actin network, we investigated lifetime of single-molecule AIP1 speckles in lamellipodia. We followed each speckle from appearance to disappearance on the images acquired at 200 ms intervals. Analysis on both N- and C-terminally tagged AIP1 yielded similar lifetime distribution of AIP1 speckles (Fig. 5A and B). Photobleaching rates of EGFP tagged probes were 0.14–0.16 s^−1^, which roughly correspond to 14% of speckle disappearance under the condition. The data after normalization for photobleaching revealed duration time distribution of F-actin binding of AIP1, which fit with a single exponential. Fitting the measurements to a single exponential yielded 1.1 s^−1^ for the dissociation rate of AIP1 from the lamellipodial actin network. The position versus lifetime plot shows no apparent variation in the average and distribution of AIP1 speckle lifetime with respect to the position from the leading edge (Fig. 5C).

This dissociation rate of AIP1 is faster than that of CP, 0.58 s^−1^ [12]. Another difference is that molecules with prolonged lifetime were less prominent in the lifetime distribution of AIP1 than in that of CP. This difference might be related to our previous observations that a portion of CP speckles had a prolonged lifetime. Our previous study noted a slow dissociation of CP along actin stress fibers. On the other hand, AIP1 shows almost no association with actin stress fibers (Fig. 1) presumably because F-actin in stress fibers is protected from cofilin by tropomyosin [43]. The difference between CP and AIP1 could therefore arise from the association of a population of CP with F-actin stabilized against the action of cofilin and AIP1 in lamellipodia. Presence of a stabilized population of actin filaments in lamellipodia has been described [7], although the underlying mechanism remains to be clarified.

### AIP1 associates evenly with F-actin network throughout lamellipodia

**Figure 6:**
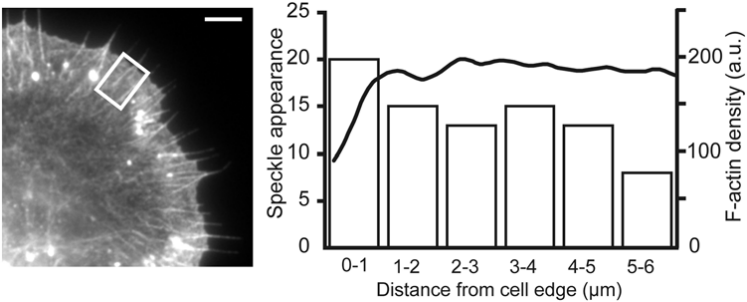
Appearance of AIP1 speckles is roughly proportional to F-actin distribution. Images of EGFP-AIP1 speckles were acquired at intervals of 300 ms for 9 s. Cells were then fixed and stained with TexasRed-phalloidin (left image). The position of newly appeared single-molecule AIP1 speckles were recorded in an area (square) devoid of dense clusters of AIP1 speckles over 25 consecutive images, n = 89. Curve, actin filament distribution measured by phalloidin binding. Columns, the number of newly-emerged AIP1 speckles in each indicated position. A representative result of 3 independent measurements is shown. Bar, 5 µm.

We next compared the emerging position of AIP1 speckles against F-actin distribution in lamellipodia. Immediately after timelapse acquisition of AIP1 speckle images at the interval of 300 ms, cells were fixed and processed for phalloidin staining to visualize F-actin distribution. We selected rectangular areas devoid of dense clusters of AIP1 speckles and aggregated F-actin for analysis. The position of appearance of single-molecule EGFP-AIP1 speckles was roughly proportional to the distribution of F-actin (Fig. 6). This flat distribution markedly contrasts with that of Arp2/3 complex, which is highly promoted in the narrow leading edge area [12]. These results suggest that cofilin-mediated filament disruption/severing occurs evenly throughout lamellipodia, irrespective of distance from the leading edge.

### AIP1-associated filament disruption is more frequent than Arp2/3 complex-catalyzed nucleation

We next measured the amount of AIP1 proteins in lamellipodia of XTC cells. AIP1 was found largely in the Triton-X 100 soluble fraction. AIP1 was estimated to consist of 0.027% of total proteins in this fraction by immunoblot analysis (Supplementary Fig. S1). We also determined the average protein content to be 2.1×10^−10^ g/cell in the Triton-X 100 soluble fraction. Distribution of AIP1 was analyzed by immunostaining with specific antibodies. We adopted ∼0.15 µm for the thickness of internal space of lamellipodia [44]. Then the concentration of AIP1 was calculated to be 1.6 µM±0.35 µM (mean±s.d.; n = 20 cells). We also estimated the concentration of Arp2/3 complex. Our estimation yielded 2.3±0.39 µM for the concentration of Arp2/3 complex in lamellipodia (mean±s.d.; n = 9 cells). From the dissociation rate of AIP1, 1.1 s^−1^ (Fig. 5) and Arp2/3 complex, 0.048 s^−1^ [12], we estimate the rate of the F-actin association of AIP1 and Arp2/3 complex-catalyzed nucleation to be approximately 1.8 µM s^−1^ and 0.11 µM s^−1^, respectively. Thus, filament network association of AIP1 is one order of magnitude more frequent than Arp2/3 complex-catalyzed nucleation in lamellipodia. We predict that a part of AIP1-associated barbed ends may initiate elongation because AIP1 does not appear to tightly cap the barbed end (See Discussion). On the other hand, the overall rate of AIP1 speckle turnover is not frequent enough to account for the high frequency severing-annealing rate that we previously hypothesized under the assumption of uniform severing throughout the actin network.

### Depletion of cofilin activity abolishes serum-stimulated actin assembly at the leading edge of XTC fibroblasts

**Figure 7:**
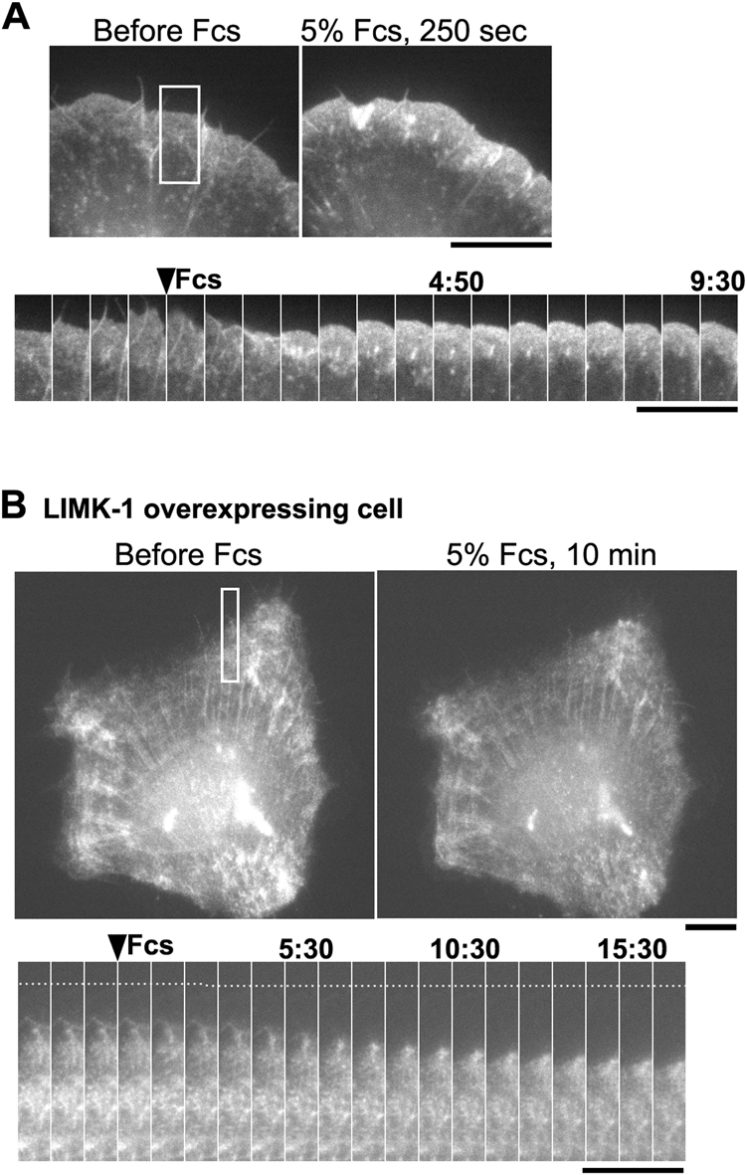
Inhibition of serum-induced lamellipod actin assembly by overexpression of hLIMK-1. (A) XTC fibroblasts were serum-starved for ∼4 h after spreading on poly-L-lysine coated coverslips and then stimulated by 5% fetal calf serum (FCS). The upper panels show EGFP-actin before and 250 s after perfusion of L-15 medium containing 5% FCS. The lower panels show time-lapse images at 40 s intervals (square). The cell edge gradually extended after serum stimulation accompanied by a marked increase in EGFP-actin fluorescence in lamellipodia. (B) The upper panels show EGFP-actin images in an XTC cell overexpressing mRFP-hLIMK-1. The lower panels are time-lapse images of EGFP-actin at the intervals of 60 s (square). Upon 5% FCS stimulation, lamellipodial F-actin structure contracts toward the cell center accompanied by a slight increase in the density of EGFP-actin. However, the total amount of EGFP-actin fluorescence associated with the peripheral actin structures was not increased. Time is in minute:second. Bars, 10 µm.

Depletion of cofilin activity abolishes the barbed-end increase at the cell periphery of metastatic MTLn3 breast carcinoma cells induced by epidermal growth factor [45]. Cofilin and its phosphatase, Slingshot play a pivotal role in lamellipod extension induced by neuregulin-1β in another breast carcinoma cells line, MCF-7 [46]. Given the frequent formation of AIP1-associated barbed ends, we wished to test the role of cofilin in stimuli-evoked F-actin assembly in our cell system. Under our observation conditions, XTC fibroblast cells are allowed to spread onto poly-L-lysine (PLL)-coated glass coverslips in the absence of fetal calf serum (FCS). The retrograde flow and density of the lamellipodial actin network become less prominent over a time course of 3 to 4 hours after cell spreading. Perfusion of L-15 medium containing 5% FCS led to a rapid increase in EGFP-actin fluorescence and the rate of retrograde flow in lamellipodia (Fig. 7A, Movie S3). In contrast, almost no induction of serum-induced cell edge actin assembly was observed in cells expressing a high amount of mRFP1-hLIMK-1 (Fig. 7B, Movie S4). Contractile movement of peripheral actin structures toward the cell center was apparent after serum stimulation, suggesting intact signaling that mediates actomyosin contraction in cells overexpressing hLIMK-1. Although it has been argued that cofilin may increase actin barbed ends not only through its severing activity [13], [45] but also by increasing the G-actin supply [23], the data (Fig. 7) demonstrate the crucial role of cofilin in stimulus-evoked lamellipod actin assembly, which is conserved between breast carcinoma cell lines and XTC fibroblasts. Our current observation of single-molecule AIP1 has now revealed the spatiotemporal dynamics of this actin reorganization process directly in lamellipodia.

## Discussion

### *In vivo* evidence of cofilin-dependent barbed end association of AIP1

The barbed end association of AIP1 has been demonstrated both biochemically and by electron microscopy [34]. This barbed end association of AIP1 requires cofilin. AIP1 is therefore thought to cap the new barbed end generated by the filament severing activity of cofilin [25], [30]. AIP1 also enhances cofilin-induced F-actin severing/disassembly. This effect is not simply caused by prevention of annealing of severed filaments by capping because other barbed end capping reagents such as gelsolin and cytochalasin D fail to enhance cofilin-induced severing [47]. This finding along with recent findings of cooperative fast disassembly of coronin-decorated F-actin by cofilin and AIP1 [35], [36] suggests that AIP1-associated filament severing/disruption and subsequent capping of the newly-formed barbed end by AIP1 may occur in a coordinated manner, although the precise relationship between these two activities remains to be solved [30], [47].

Our current analysis adds *in vivo* evidence of cofilin-dependent barbed end association of AIP1. First, the density of AIP1 speckles is negatively correlated with the expression level of mRFP1-tagged LIMK1. Second, the formation of AIP1 speckles is rapidly abolished upon Jas treatment concomitantly with disappearance of cofilin speckles. These two findings indicate that cofilin-assisted filament severing/disruption is prerequisite for the robust association of AIP1 with F-actin. Third, the decay rate of persistent AIP1 speckles becomes significantly slower upon Jas treatment, which is analogous to the response of another barbed end binding molecule, CP. These lines of evidence support our interpretation that AIP1 speckles represent their interaction with barbed ends generated by cofilin-catalyzed filament severing or the ‘burst’ mode of fast filament disruption proposed by Kueh et al. [36].

Our data also support our previous study. We previously attributed the fast dissociation of capping protein (CP) from the actin network to cofilin-induced F-actin severing [12]. This interpretation was based on three observations of attenuation of CP dissociation induced by jasplakinolide (Jas), LIMK1 overexpression and stress fiber association of CP probes. The experiments employing LIMK1, which phosphorylates and inactivates cofilin [24], directly support dependency of CP dissociation on the activity of cofilin. Our new data confirm the ability of Jas to prevent cofilin from interacting with F-actin. Furthermore both endogenous and EGFP-tagged cofilin show scarce association with actin stress fibers in XTC cells. F-actin in stress fibers is decorated with tropomyosin which may protect F-actin from cofilin [43]. These findings strengthen our previous interpretation that the activity of cofilin is responsible for the fast dissociation of CP from the actin network.

### AIP1 turnover rate is insufficient to account for frequent severing-annealing theory

Supported by the above notions, our current analysis determined the frequency of AIP-associated filament disruption to be 1.8 µM s^−1^ in lamellipodia. If we assume ∼1000 µM for the F-actin concentration [44], the rate of AIP1 binding to F-actin corresponds to a frequency of 1.8×10^−3^ s^−1^ or 1 s^−1^ per ∼560 subunits long filament.

The size of actin oligomers, if they exist, required to diffuse off the actin network is currently unknown. Detailed electron microscopy [48] demonstrates that the intercross between filaments occurs every 14–16 nm on average which corresponds to ∼5 actin subunits. The detergent-extracted dendritic nucleation network in the actin comet tail of *Listeria monocytogenes* in macrophages largely consists of short filaments less than 0.3 µm (typically ∼0.1 µm; Figure 8 in [49]). From these observations, the size of diffusible actin oligomers can be estimated to be within the range between 5 and 30 subunits. To achieve fragmentation of CP-bound F-actin to these sizes, a severing rate of 0.1∼0.02 s^−1^ is required. Hence the determined AIP1-mediated filament severing rate, 1.8×10^−3^ s^−1^, falls short of the severing rate predicted in our frequent filament severing-annealing hypothesis at least by one order.

**Figure 8:**
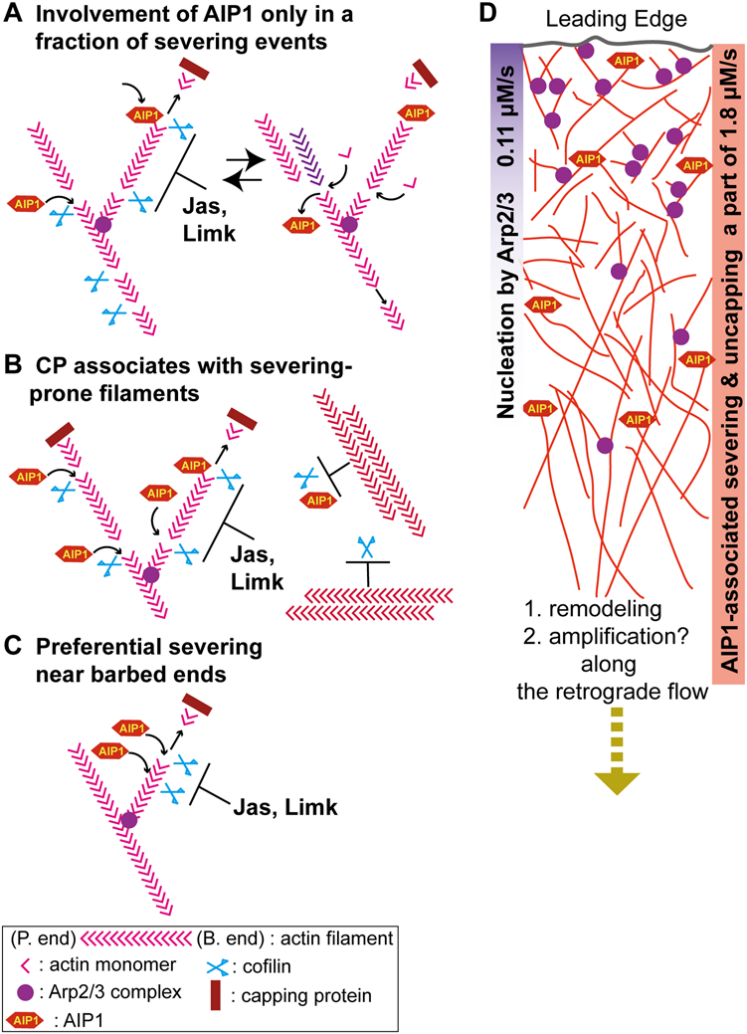
Models for mode and function of AIP1-associated filament disruption. (A) AIP1 may be associated with only a fraction of filament severing events within the network. (B) CP may preferentially associate with the dynamic filaments prone to AIP-mediated filament severing/disruption. We estimate that roughly a half of F-actin is stabilized in lamellipodia of XTC cells. (C) Alternatively, AIP1-associate filament severing/disruption may occur preferentially at the proximity of the barbed end within a single filament. (D) Cooperation of nucleation and filament severing/disruption to facilitate leading edge protrusion. Release of free barbed ends from AIP1-associated filament disruption processes may amplify the Arp2/3 complex-initiated actin nucleation increase at the leading edge by repeatedly acting along the retrograde flow. The severing and end-to-end annealing may also be relevant to actin remodeling activities that confer filament angle and length alternation along the aging of network seen in published electron micrographs [10], [48].

Several explanations may account for this discrepancy. AIP1 might be involved only in a fraction of filament severing events (Fig. 8A). *In vitro*, the inhibitory effect of AIP1 on cofilin-evoked F-actin growth corresponds well with the degree of filament fragmentation induced by AIP1 [34]. Also during filament disassembly exerted by cofilin, coronin and AIP1, barbed ends are protected from CP [36]. These observations are best explained by robust association of AIP1 with newly-formed barbed ends after filament disruption/severing. We therefore anticipate detecting a large fraction of AIP1-associated filament disruption events by our single-molecule observation of AIP1. However, the possibility that AIP1 does not participate in all severing reactions cannot be excluded.

Another mechanism that could compensate for the discrepancy in part is preferential association of CP with severing-prone filaments (Fig. 8B). Detailed speckle analysis by us [6] and others [7] has detected a wide distribution of F-actin lifetime in lamellipodia. Existence of long-lived F-actin implies stabilization of a fraction of F-actin by certain mechanisms. In this regard, it is noteworthy that a minor fraction of CP speckles have a prolonged retention time [12]. Because CP speckles bound to actin stress fibers, to which cofilin and AIP1 scarcely associate, are stabilized in the lamellae region, CP might be stabilized analogously when bound to filaments resistant to cofilin also in lamellipodia. We therefore postulate that the majority of CP displaying fast dissociation (∼0.58 s^−1^) binds the dynamic population of F-actin prone to cofilin-catalyzed disassembly. We recalculated the previous F-actin lifetime distribution data [6] by weighing lifetime of each population to assess lifetime distribution of F-actin existing at a given moment. The stabilized population with >100 sec filament lifetime turned out to constitute a half (46%) of F-actin in lamellipodia of XTC cells. We can thus re-estimate that AIP-associated severing occurs at ∼1 s^−1^ per 280 subunits in the dynamic population of F-actin.

Finally, the filament near the barbed end may be more prone to AIP1-associated filament disruption than the other portion of the filament (Fig. 8C). This model is in agreement with the recent biochemical studies showing fast actin disassembly at both barbed and pointed ends by coronin, cofilin and AIP1 [35], [36]. The overall filament severing rate might not be as frequent as postulated in our frequent filament severing-annealing hypothesis. Notably, overall AIP1 turnover occurs at 1.8 µM/s, which appears to roughly correspond to the overall dissociation frequency of CP from the network [12]. Given the much slower disassembly rate of the pointed-end side of filaments in lamellipodia (the dissociation rate of Arp2/3 complex, 0.048 s^−1^ [12]), it is tempting to speculate that cellular actin filaments might undergo one filament-end (barbed-end) dynamicity analogous to microtubules rather than filament treadmilling. Further extensive studies will be required to challenge this intriguing possibility.

### Caution in the interpretation of F-actin turnover data between single-molecule speckle observation and FRAP analysis

A recent study employing FRAP analysis using EGFP-actin [8] claims that FRAP data showing slow recovery of the photobleached actin are inconsistent with our speckle analysis data showing a large fraction of short-lived F-actin [6]. Taking this opportunity, we discuss how such discrepancy might have arisen between two different approaches. Firstly, it is of note that single-molecule speckle analysis of actin, which measures F-actin lifetime per assembly event [6], detects rapidly depolymerizing F-actin species repeatedly within the observed time window. By contrast, FRAP (fluorescence recovery after photobleaching) analysis measures the recovery kinetics of the label existing at a given moment. Hence direct comparison between F-actin lifetime distribution data from speckle turnover analysis and the decay curve in FRAP experiments may not be valid. As mentioned in the last section, our previous speckle analysis can be reinterpreted as showing that nearly a half of F-actin existing at a given moment is estimated to have extended lifetime (>100 sec) in lamellipodia of XTC cells. In the situation where a half of F-actin is stabilized, the decay of FRAP labels still looks similar to single exponential decay and its apparent decay rate could become roughly one-third of the decay rate of the dynamic F-actin species if one fits the data with a single exponential. The simulation employed in the above FRAP study [8] does not consider the presence of mixed populations of F-actin with different disassembly rates.

Secondly, as already proven by mathematical analysis [50], fast reincorporation of dissociated labels into the same photobleached area retards FRAP kinetics substantially slower (∼by half) than the true actin disassembly rate [6], [50]. Thus FRAP analysis could be prone to underestimate the turnover rate of rapidly disassembling F-actin species for these two reasons. For this purpose, single-molecule observation has the considerable advantage over FRAP because of superior spatiotemporal resolution.

Thirdly, if our severing-based actin release model [12] applies, actin disassembly may in part proceed through the release of slowly-diffusing actin oligomers, which would further slow released actin diffusing out of the photobleached area. This third mechanism is intriguing, but has not been proven directly.

On the other hand, we also need to point to a problem of our previous speckle analysis [6]. The experiment design was not optimal for detecting the stabilized F-actin population. Due to inefficient optics and the resultant fast photobleaching of EGFP-actin, we previously set the duration to detect newly appearing actin speckles for 100 sec (and followed them until they disappeared). This duration may not be enough to precisely capture actin speckles with lifetime longer than ∼50 sec. Thus, while we are confident in our previous conclusion of rapid turnover of the large population of newly-polymerized actin in lamellipodia, the precise size of the stabilized F-actin population remains to be examined also in XTC cells. We hope to revisit this issue of the difference between FRAP and single-molecule speckle analyses elsewhere because elucidating dynamics of molecular complex formation and disassembly is becoming more important in biology.

### Possible division of labor between Arp2/3 complex-catalyzed nucleation and filament severing

An unsolved question is whether AIP1 dissociates rapidly enough to allow barbed end growth after its association to filament ends. During actin disassembly induced by AIP1, cofilin and coronin, barbed ends appear to be protected from CP [36]. However, this protection is not strict as a high concentration of profilin∶actin complex blocks disassembly of *Listeria* actin comet tail by ∼50% at 8 µM and by >80% at 16 µM [35]. AIP1 dissociates rapidly from barbed end (τ<10 s) [36]. Consistently, our data show that AIP1 dissociates from barbed end at 0.1∼0.2 s^−1^ in jasplakinolide-treated cells where severing/disruption-based release of CP is attenuated severely [12]. If this reflects spontaneous dissociation of AIP1, 10∼20% of AIP1 dissociation events may lead to the formation of free barbed ends. Our data show that AIP1 speckles emerge throughout lamellipodia, whereas the emergence position of Arp2/3 complex speckles is heavily biased toward the 0.65 µm wide lamellipodium tip area [12]. Given ∼15 times faster turnover of AIP1 than that of Arp2/3 complex, we predict that elongation of disassembly-generated barbed ends after occasional release of AIP1 could quantitatively account for the ubiquitous actin polymerization [5], [6], [7] and free barbed ends [12], [51] in the body of lamellipodia.

Arp2/3 complex receives signals from cascades downstream of Rac-WAVE/Scar and Cdc42-WASP signaling [52] and adds new filament nuclei at the lamellipodium tip. At the back of the leading edge, AIP1-associated barbed end generation can serve repeatedly along the retrograde flow and amplify the filament mass. We thus postulate a division of labor in which filament severing/disruption may function to amplify nucleation signals initiated by Arp2/3 complex (Fig. 8D). In accordance with this idea, serum stimulation fails to promote actin assembly at the cell periphery of XTC cells depleted of cofilin activity.

In summary, our study revealed fast association and dissociation behavior of AIP1 with the lamellipodial actin network, which represents its barbed end association during cofilin-dependent actin disruption. Together with fast dissociation of CP [12], our work highlights fast actin remodeling involving filament severing/disruption quantitatively in the body of lamellipodia. Modeling of spatiotemporal actin remodeling processes including severing and end-to-end annealing reactions will be a challenge but crucial to understand how such actin remodeling processes propagate within the actin array and regulate cell edge protrusion.

## Methods

### Plasmids

The expression vectors for EGFP-actin and mPlum-hLIMK1 were described [6], [12]. *Xenopus* AIP1 cDNA (GenBank accession number, BC077202) was obtained from IMAGE consortium [38]. The expression constructs for AIP1 and *Xenopus* cofilin, XAC2 [40], were generated by introducing AIP1 or XAC2 cDNA into either EGFP or mPlum [37] expression vector harboring the defective CMV promoter [6].

### Live cell imaging and fluorescent speckle microscopy

Live cell imaging was carried out as described [6], [12]. Briefly, XTC cells were maintained in 70% L-15 Leibovitz medium (Invitrogen) supplemented with 10% fetal calf serum (FCS). Cells were transfected using Superfect (Qiagen) and maintained after passage into fresh flasks. Before experiments, cells were trypsinized and allowed to spread on a poly-L-lysine (PLL)-coated glass coverslip attached to a flow cell in 70% L-15 medium without FCS. The flow cell was placed on the stage of an Olympus BX52 microscope equipped with a cooled CCD camera (Cool SNAP HQ, Roper Scientific), an Olympus BX51 microscope equipped with Cascade II:512 (Roper Scientific) or an Olympus inverted IX71 microscope equipped with UIC-QE (Molecular Devise). Time-lapse imaging was carried out at 21–23°C using the Metamorph software (Molecular Devise) up to 120 min after cells were seeded. Fluorescent speckle microscopy was carried out by observing cells expressing a low amount of EGFP-tagged proteins using an objective, PlanApo 100× (NA 1.40) (Olympus). A restricted area near the cell edge was illuminated using a 75 W xenon illumination system. Speckle lifetime measurement was carried out by tracking individual speckles manually. The photobleaching rate of EGFP-probes was measured by illuminating entire cell areas under the identical condition on the day of each experiment. The method of normalization of data for photobleaching was described [6].

### Antibody and immunocytochemistry

The rabbit anti-AIP1 antibodies were raised against recombinant glutathione S-transferase (GST)-tagged AIP1 expressed in *E. coli*. Antibodies reactive to the GST tag were depleted by incubating with an excess amount of irrelevant GST-fusion proteins immobilized on nitrocellulose membranes. The anti-XAC2 antibodies were raised against recombinant XAC2 cleaved from GST purified as previously [40]. For immunocytochemistry, cells were fixed with 3.7% paraformaldehyde in Cytoskeleton Buffer (10 mM MES, [pH 6.1], 90 mM KCl, 3 mM MgCl_2_, 2 mM EGTA, 0.16 M sucrose) for 20 min at room temperature, and then permeabilized in phosphate buffered saline containing 0.1% Triton X-100. Texas Red-X phalloidin (Invitrogen) was used for staining F-actin. Cy2 anti-rabbit IgG and Cy3 anti-rabbit IgG (Jackson ImmunoResearch Laboratories) were used as a secondary antibody.

## Supporting Information

Figure S1 Quantification of AIP1 and Arp2/3 complex in lamellipodia. The amount of AIP1 (A) and the Arp2/3 subunit, p40 (B) was quantified by immunoblot analysis using recombinant proteins as standards (left panels). The fraction of proteins localized in the peripheral area of lamellipodia (between two circles) was estimated from fluorescence intensity of immunostaining (right panels). For the analysis on AIP1 (A), two specific anti-sera raised against GST-AIP1 were used after depleting antibodies against the GST tag by incubating with a large excess of irrelevant GST-fusion proteins blotted onto nitrocellulose membranes. For p40 (B), specific antibodies were affinity-purified from anti-sera against 6xHis-tagged p40 [12] using immobilized GST-tagged p40. Bars, 5 µm.
(0.78 MB TIF)

Movie S1 mPlum-AIP1 (red) and AIP1-EGFP (green) in XTC cells. Time is in second.
(2.19 MB MOV)

Movie S2 Single-molecule AIP1-EGFP speckles acquired at 500 ms intervals. Time is in second.
(0.28 MB MOV)

Movie S3 XTC fibroblasts expressing EGFP-actin were serum-starved for 4 h after spreading on poly-L-lysine coated coverslips and then stimulated by 5% fetal calf serum (FCS). Images of EGFP-actin are shown. Time is in minute:second. Bar, 5 µm.
(1.41 MB MOV)

Movie S4 An XTC cell overexpressing mRFP-hLIMK-1 was stimulated by 5% FCS. Images of coexpressed EGFP-actin are shown. Time is in minute:second. Bar, 5 µm.
(0.46 MB MOV)
