# Supplementary figures and images for: An Order of Magnitude Faster AIP1-Associated Actin Disruption than Nucleation by the Arp2/3 Complex in Lamellipodia
Source: PLoS One. 2009 Mar 17;4(3):e4921. doi: 10.1371/journal.pone.0004921 (PMC2654150; doi:10.1371/journal.pone.0004921)

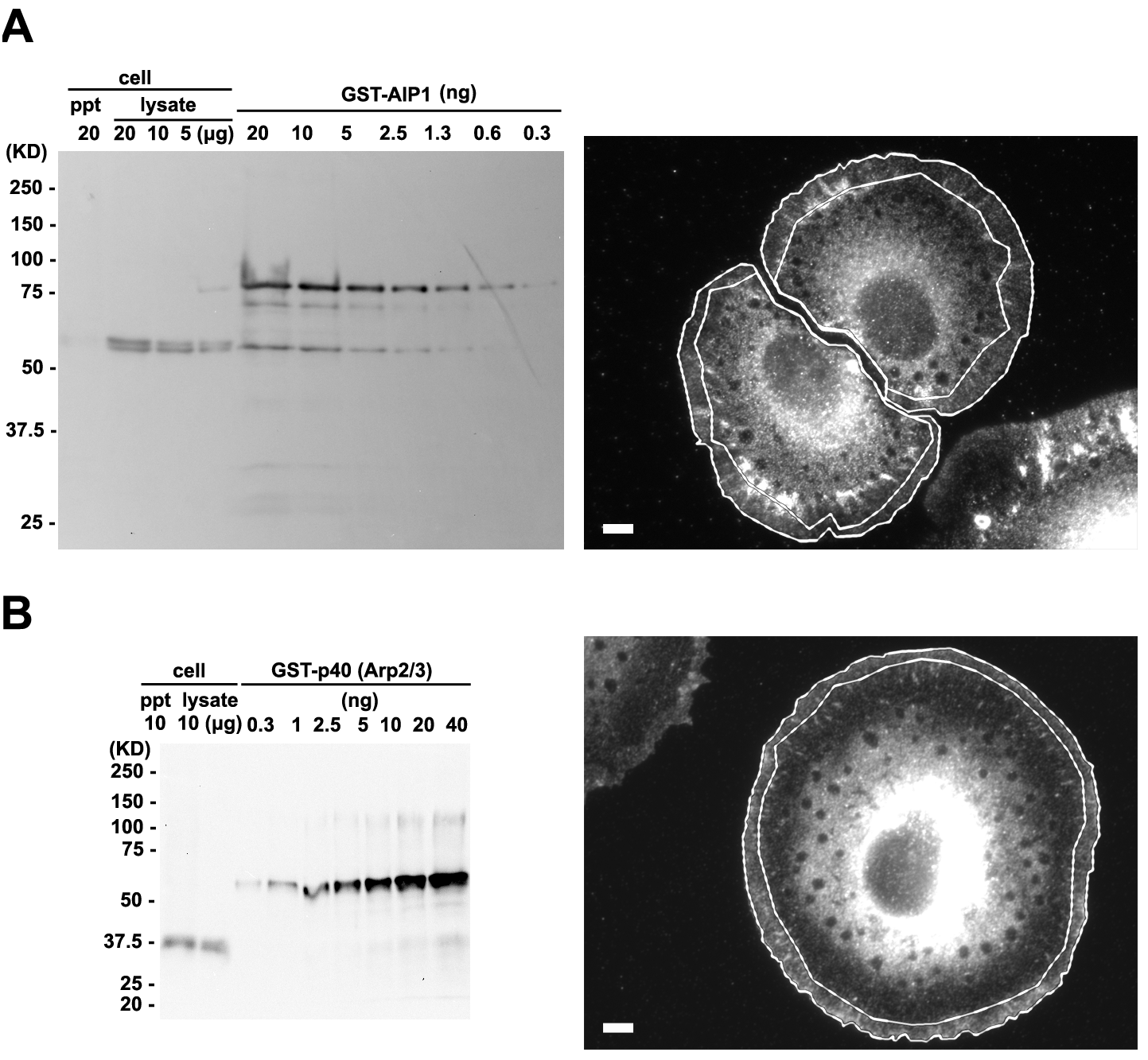

Supplement: Figure S1 — Quantification of AIP1 and Arp2/3 complex in lamellipodia. The amount of AIP1 (A) and the Arp2/3 subunit, p40 (B) was quantified by immunoblot analysis using recombinant proteins as standards (left panels). The fraction of proteins localized in the peripheral area of lamellipodia (between two circles) was estimated from fluorescence intensity of immunostaining (right panels). For the analysis on AIP1 (A), two specific anti-sera raised against GST-AIP1 were used after depleting antibodies against the GST tag by incubating with a large excess of irrelevant GST-fusion proteins blotted onto nitrocellulose membranes. For p40 (B), specific antibodies were affinity-purified from anti-sera against 6xHis-tagged p40 [12] using immobilized GST-tagged p40. Bars, 5 µm. (0.78 MB TIF) [file pone.0004921.s001.tif]
